# Hot chocolate: pre-warming chocolate agar improves correct organism identification from choc drop by 16.1%

**DOI:** 10.1099/jmm.0.002057

**Published:** 2025-09-02

**Authors:** Mark Fahmy, Mark Armstrong, Nicola Townell

**Affiliations:** 1 Microbiology, Pathology Queensland, Royal Brisbane and Women’s Hospital, Brisbane, QLD 4006, Australia; 2 Microbiology, Mater Pathology, Mater Hospital Brisbane, South Brisbane, QLD 4101, Australia; 3 Faculty of Medicine, University of Queensland, Herston, QLD 4006, Australia; 4 Department of Infectious Diseases, Mater Public Hospital Brisbane, South Brisbane, QLD 4101, Australia

**Keywords:** blood culture, choc drop, matrix-assisted laser desorption/ionization time-of-flight MS (MALDI-TOF MS), rapid diagnostics, sepsis

## Abstract

**Introduction.** Blood cultures are an important test for the diagnosis of sepsis, and the time to an accurate result can allow for optimized antibiotic therapy. The ‘choc drop’ is a commonly performed, reliable and cheap rapid identification method, using a drop of positive blood culture broth incubated for a short period (2–4 h) before performing matrix-assisted laser desorption/ionization time-of-flight MS on any early growth/microbiological veil present.

**Hypothesis.** Due to the short incubation time (~2 h), agar plates may not reach the target temperature for optimal bacterial growth, leading to reduced accuracy of identification. Pre-warmed agar plates may allow for target temperature attainment more rapidly and therefore lead to more accurate identification in short-term incubation.

**Aim.** To investigate if pre-warmed chocolate agar used for choc drop identification resulted in increased rates of correct identification.

**Methodology.** In this study, baseline performance of choc drop identification using room temperature chocolate agar plates (*n*=542) was compared with an interventional period of pre-warmed chocolate agar plates (*n*=426).

**Results.** Pre-warmed plates showed an increase in correct identification rate of 16.1% [95 % confidence interval (CI) 9.8%–22.4%, *P*=0.0013], largely due to a 17.1% (95% CI 11.1%–23.1%, *P*<0.0001) decrease in Gram-positive bacteria returning ‘no identification’. There were slightly more errors in the interventional (*n*=11, 2.8%) compared with the baseline period (*n*=2, 0.2%).

**Conclusions.** Pre-warming plates appears to be a cheap, effective and easy to implement method for improving the diagnostic yield of short-term incubation for rapid blood culture identification.

## Introduction

The blood culture is an extremely commonly ordered microbiological investigation and remains the gold standard for the diagnosis of bacterial sepsis [1]. Advances in automation (such as automated incubators/detection systems) have improved the diagnostic yield and time to a positive result for blood cultures [2]. However, once a positive culture has been identified, there are still important areas of potential delay that can impact clinical outcomes, such as time to identify more resistant organisms, which require a change from empirically selected antimicrobials [3].

One of the key factors associated with improved survival in sepsis is a shorter time to appropriate antibiotic therapy initiation [4]. This itself depends on high-quality, accurate and rapid laboratory identification of organisms isolated from positive blood cultures, which has in turn led to a number of routine laboratory practices such as telephoning results of Gram stains directly to clinicians, direct and rapid antibiotic susceptibility testing [5], as well as a variety of methods for rapid identification of organisms directly from blood culture broth. One of the most frequently used methods is the incubation of a drop of blood culture broth onto a plate of non-selective media (usually chocolate agar) for a short period of time (usually between 2 and 6 h) before performing matrix-assisted laser desorption/ionization time-of-flight MS (MALDI-TOF MS) identification on any early growth/microbial veil present [6]. This method is often referred to as the ‘choc drop’ and is a routine laboratory practice globally and is often a standard part of many laboratories’ blood culture workflow.

The choc drop is a reliable and cheap method for preliminary identification with a concordance rate with final identification somewhere between 64 and 80.47% [6,7]. Various methods for enhancement of early identification have also been described, including centrifugation of blood culture broth and performance of direct MALDI-TOF MS identification on the resulting bacterial pellet [8], but these methods are not yet widely used and require increased handling, scientist time and consumables.

There are a number of factors that are well recognized to result in better MALDI-TOF MS identification of bacteria, some of which are technical (e.g. proper technique/inoculum used on MALDI-TOF MS plates and use of correct amount of matrix) and others of which relate to sufficient growth of micro-organism to allow for correct identification (correct media, lack of inhibitory substances/antibiotics, correct atmosphere and temperature for incubation). Temperature in particular is an interesting and potentially underappreciated factor that may impact rates of bacterial growth, especially for short-term incubation such as is used for the choc drop.

There are limited studies that directly assess the effect of incubator temperature on rates of bacterial growth and successful identification of bacteria from blood cultures (or other body sites), but it has been experimentally demonstrated that there is variability in time to achieve stable temperature depending on the position of an agar plate in the incubator and within a stack of other plates. Of interest, one study demonstrates that it can take up to 1.5–2.5 h to reach incubation temperature depending on the position within a stack of plates [9]. As the period of incubation may be as little as 2 h, it is possible that the plate conditions for growth remain sub-optimal for a significant period of the choc drop incubation, which may reduce the chance of correct identification.

The aim of this study was to evaluate if pre-warmed chocolate agar would improve rates of correct early identification via the choc drop compared with the previous standard laboratory practice of using room temperature agar. While pre-warmed agar has previously been described as part of some laboratories’ workflow [10], there has not yet been a study comparing pre-warmed and non-pre-warmed agar plates for early identification of positive blood cultures.

## Setting

This study was carried out at Mater Pathology, a medium-sized laboratory servicing several tertiary and secondary level public and private hospitals with a wide range of specialist departments including Intensive Care Unit, surgical and haematology/oncology populations providing care to adult and maternal and paediatric/neonatal populations. In 2024, a total of 20,221 blood cultures were received, of which 1,815 were positive. One hundred sixty-five (9.1% of positive cultures) were deemed to be contaminants after microbiologist review.

## Methods

The standard protocol for all positive blood cultures (as identified by the BACT/ALERT® Virtuo® automated system and confirmed by Gram stain) at our laboratory is for setup of a choc drop plate [11] with one drop of the blood culture broth being placed on the centre of chocolate agar plate, which is then incubated in 5% CO_2_ at 37 °C. Twice a day (at 5 PM and 6 AM), any choc drop plates that have had a minimum 2 h of incubation time are placed onto the MALDI-TOF MS (VITEK MS® v 3.2; bioMerieux) for identification with a positive control spot of ATCC *8379 Escherichia coli* applied to the slide by the scientist. The chocolate agar plates are all manufactured in-house and have completed relevant quality control requirements on each new batch. Plates are refrigerated prior to use. There is a supply of room temperature plates in use at the microscopy/setup bench.

MALDI-TOF MS is performed regardless of visible growth, and the results including the identification of the organism are documented in the laboratory notes for review by the microbiologist. Confidence of identification is also noted, with ≥99% being considered a high confidence identification and <99% considered a low confidence identification. This preliminary result is not released as part of the current reporting practices but may be telephoned to a clinician at the discretion of the reporting microbiologist (e.g. *Staphylococcus aureus* to ensure prompt empiric treatment and investigation for complicated infection is initiated).

Final identification is usually performed from an isolated colony on day 1 growth incubated on routine agar (blood/chocolate agar, anaerobic blood agar for anaerobic blood cultures and either Columbia colistin-nalidixic acid, MacConkey or Sabouraud dextrose agar depending on the Gram stain), which is usually achieved via MALDI-TOF MS, excluding fastidious or slow-growing organisms where additional methods (e.g. VITEK-2®; bioMerieux, Analytical Profile Index; bioMerieux, or rarely by 16S rRNA or whole-genome sequencing) may be required.

For the purposes of the study, a period of retrospective baseline data collection of choc drop data was undertaken. A 10% difference in first-time correct, high confidence identification was taken as an estimated significant change between methods. Power calculations suggested that a sample size of at least 730 identifications was required. Data collected included the choc drop identification, the final identification and the confidence of each identification as a percentage.

Once this baseline data was collected, a change was made to the standard protocol to inoculate all choc drops onto pre-warmed chocolate agar. This was achieved by placing a stack of ten chocolate agar plates in a walk-in incubator (temperature 35–37 °C) for 12 h prior to the start of the testing period. To ensure that all future plates would have had sufficient time to warm, scientists performing a choc drop would select the plate from the top of the stack, before replacing that plate with a new one placed at the bottom of the stack. This was also done to ensure that the agar was not left for a long period in the incubator, which could lead to excessive drying out or potential growth of contaminating organisms.

Standard laboratory practices, methods of identification, instruments used and reporting conventions remained the same during the study period as in the baseline period. The data collected during the intervention period was the same as that for the baseline data collection. Statistical calculations including 95% confidence intervals (CIs) and two-tailed *P*-values were calculated for percentage changes between the baseline and intervention periods as a measure of statistical significance.

## Results

### Baseline period

During the baseline period, a retrospective review of 542 blood cultures with choc drops was performed, which was collected over a 4-month period (Table 1). Thirty-five (6.4 %) blood cultures were polymicrobial and therefore excluded from the analysis. Of the remaining monomicrobial blood cultures, 203 (38.9%) showed no identification by choc drop, 275 (52.7%) showed a correct, high confidence identification (≥99%) by choc drop compared with final identification and 27 (5.2%) showed a correct, low confidence identification (<99%).

**Table 1.** Comparison of blood cultures by concordance of choc drop identification with final identification, type of blood culture bottle and organisms isolated in the baseline and intervention periods

	Baseline period	Intervention period
Blood cultures with choc drop and final identification	542	426
Polymicrobial blood cultures (excluded from analysis)	35 (6.4%)	29 (6.8%)
Monomicrobial blood culture bottles with choc drop	507	397
**No identification by choc drop**	203 (40.0%)	91 (22.9%)
**No identification organism type:**		
Gram-positive	153 (75.4%)	61 (67.0%)
Gram-negative	35 (17.2%)	26 (28.6%)
Yeast	15 (7.4%)	4 (4.4%)
Blood culture bottle type – no ID:		
Aerobic	102 (47.7%)	39 (42.8%)
Anaerobic	68 (31.8%)	40 (44%)
Both	33 (15.4%)	12 (13.2%)
**Correct, high confidence identification (≥99%) by choc drop**	275 (54.2%)	279 (70.3%)
**Correct, high confidence identification (≥99%) organism type:**		
Gram-positive	145 (52.9%)	157 (56.3%)
Gram-negative	120 (43.8%)	122 (43.7%)
Yeast	5 (1.8 %)	0 (0%)
Blood culture bottle type – correct ID:		
Aerobic	131 (47.0%)	117 (41.9%)
Anaerobic	82 (29.4%)	76 (27.2%)
Both	73 (26.2%)	86 (30.8%)
**Correct, low confidence identification (<99%**)	27 (5.2%)	16 (4.0%)
**Incorrect identification**	2 (0.4%)	11 (2.8%)

There were two (0.4%) incorrect identifications in the baseline period (Table 2). The first instance was a choc drop identification of *Klebsiella pneumoniae* (99.9% confidence), when final identification was *Escherichia coli* (final identification confirmed by MALDI-TOF MS from day 1 growth on an isolated colony as well as motility, indole positivity and VITEK-2 identification). The second was a choc drop identification of *Raoultella ornithinolytica* (98.8% confidence), which was incongruent with the Gram stain (Gram-positive cocci in chains) and the final identification of *Streptococcus lutetiensis*.

**Table 2.** Detailing incorrect choc drop identification compared with final identification in the baseline and intervention periods

Baseline period	Choc drop identification	Final identification	Gram stain congruence	Clinical significance	Error classification
1.	*R. ornithinolytica* (98.9% confidence)	*Streptococcus lutetiensis*	Gram stain incongruent	Low	Incorrect identification
2.	*K. pneumoniae* (99.9% confidence)	*Escherichia coli*	Gram stain congruent	Low	Incorrect identification
**Intervention period**	**Choc drop identification**	**Final identification**	**Gram stain congruence**	**Clinical** **significance**	**Error classification**
1.	*Staphylococcus epidermidis* (99.9% confidence)	*Micrococcus luteus*	Gram stain congruent	Low	Incorrect identification
2.	*Enterococcus faecalis/Vibrio fluvialis* (49%/50% confidence)	*Streptococcus* sp.	Gram stain congruent	Low	Low confidence/multiple organism identification
3.	*Streptococcus suis/Staphylococcus haemolyticus* (50%/50% confidence)	*Clostridium* sp.	Gram stain incongruent	High (potential under treatment)	Low confidence/multiple organism identification
4.	*Vibrio fluvialis* (99.9% confidence)	*Staphylococcus epidermidis*	Gram stain incongruent	High (potential over treatment)	Incorrect identification
	*Enterococcus faecalis/Vibrio fluvialis* (45.7 %/54.2% confidence)	*Propionibacterium acnes*	Gram stain incongruent	Low	Low confidence/multiple organism identification
	*Streptococcus intermedius* (50% confidence)	*Staphylococcus capitis*	Gram stain incongruent	Low	Low confidence organism identification
	*Escherichia coli* (99.9% confidence)	*Staphylococcus capitis*	Gram stain incongruent	High (potential over-treatment)	Incorrect identification
8.	*Staphylococcus capitis/ Klebsiella aerogenes/Vibrio fluvialis/Enterococcus faecalis* (26.9%/26.8%/26.9%/19.3% confidence)	*Staphylococcus capitis*	Gram stain congruent	Low	Low confidence/multiple organism identification
9.	*Staphylococcus capitis* (99.9% confidence)	*Bacteroides fragilis*	Gram stain incongruent	High (potential under treatment)	Incorrect identification
	*Streptococcus parasanguinis* (99.9% confidence)	*Streptococcus mitis/oralis*	Gram stain congruent	Low	Incorrect identification
11.	*Staphylococcus aureus* (99.9% confidence)	*Staphylococcus hominis*	Gram stain congruent	High (potential over-treatment)	Incorrect identification

## Intervention period

During the intervention period, a prospective collection of 426 blood cultures with choc drops was performed, which was collected over a 4-month period (Table 1). Twenty-nine (6.8%) blood cultures were polymicrobial and therefore excluded from the analysis. Of the remaining monomicrobial blood cultures, 91 (22.9%) returned no identification by choc drop, 279 (70.3%) returned a correct, high confidence identification and 16 (4.0%) returned a correct, low confidence identification. Eleven (2.8%) returned an incorrect identification.

There were 11 (2.8%) incorrect choc drop identifications during the intervention period (Table 2). Of these, six were of organisms with a Gram status incongruent with the initial Gram stain, and five were congruent with the initial Gram stain. Five results were also notable for being low confidence identifications, or identifications suggesting multiple organisms.

## Comparison between baseline and intervention periods

As detailed in Table 1, the proportions of blood culture bottle types included in the analysis were similar between the aerobic (47.7% vs 42.8%), anaerobic (31.8% vs 44 %) and both (15.4% vs 13.2%) bottles.

The rate of correct, high confidence identification from choc drop in the baseline period was 54.2%, and the rate of correct, high confidence identification from choc drop in the intervention period was 70.3%, suggesting an increase in correct identification rate of 16.1% (95% CI 9.8%–22.4%, *P*=0.0013) meeting both the pre-specified increase of 10% and being statistically significant. There appeared to be a significant reduction of 17.1% (95% CI 11.1%–23.1%, *P*<0.0001) in the proportions of blood cultures returning no identification between the baseline and intervention periods, suggesting that this was likely the major contributor to the improvement in test performance.

**Fig. 1.**
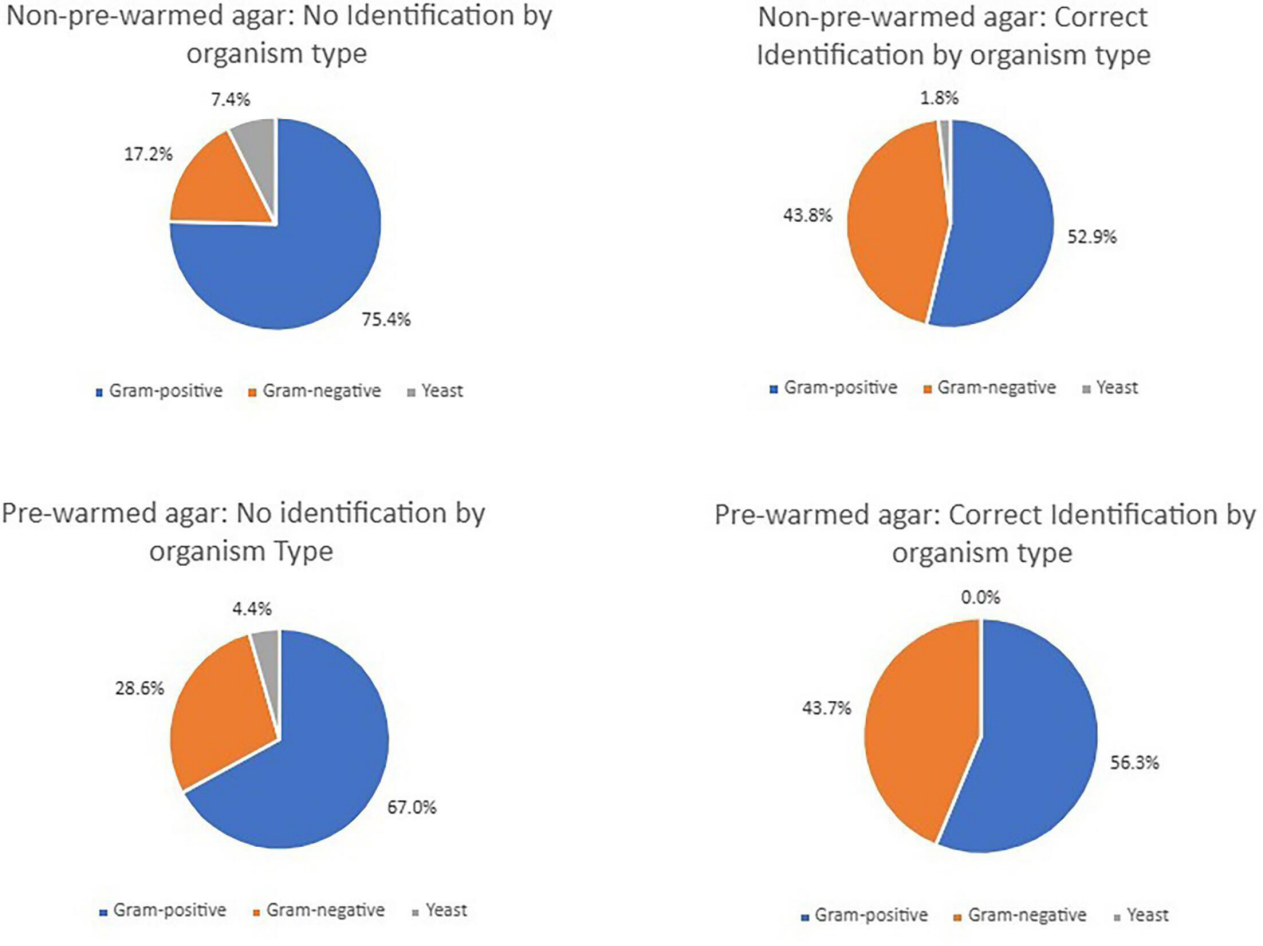
Pie charts showing proportions of blood cultures in the no identification compared with the correct identification groups in the baseline and intervention periods.

There were some differences between the types of organisms isolated in the baseline and intervention period (Table 1), with slightly more yeasts being isolated in the baseline period (4.2%, *n*=20) compared with the intervention period (1%, *n*=4), but it is notable that the baseline choc drop was able to identify only a small minority of yeasts (all *Pichia kudriavzevii n*=5). A full list of organisms isolated during the baseline and intervention periods is summarized in Supplementary Table 1. Rates of Gram-positives and -negatives were largely similar between the two periods (Fig. 1).

As summarized in Table 2, the rate of errors (incorrect choc drop ID compared with the final result) was higher in the intervention period (*n*=11, 2.8%) compared with the baseline period (*n*=2, 0.2%). This difference was small at 2.6% (95% CI 0.9%–4.3%, *P*=0.0025) but reached statistical significance. As summarized in Table 2, of the 11 errors observed during the intervention period, 6 were assessed as having low clinical significance. These involved either the misidentification of low virulence or likely contaminant organisms, or organisms typically covered by a similar spectrum of empiric therapy. Of the remaining five errors deemed potentially more clinically significant, due to the risk of inappropriate treatment escalation or de-escalation, four were unlikely to have had any clinical impact. In these cases, the preliminary identification was inconsistent with the initial Gram stain findings, and standard testing algorithms would generally preclude reporting such discrepancies. The only error of note would be the single identification of *Staphylococcus aureus* via choc drop which transpired to be definitively identified as *Staphylococcus hominis*. This error may have led to over-investigation or treatment, but it is notable that all choc drop identifications are reported as preliminary and may be subject to change or revision when results of identification from a single colony are available.

## Discussion

The findings suggest that pre-warming chocolate agar for rapid choc drop identification of organisms isolated in positive blood cultures resulted in more frequent correct, high confidence identifications. This is an important finding as the implementation of this process is simple and cheap and requires no extra consumables or specialized handling, in contrast to other described methods for rapid blood culture identification, which rely on molecular methods [12] or specialized handling requirements [13]. It is also easily adaptable to any lab setting already carrying out choc drops as part of their routine blood culture process.

The footprint of change is also extremely small, with the only additional impact on incubator space being a small rack of chocolate agar plates. We did not observe any increase in contaminating organisms before inoculation or excessive drying of chocolate agar during the study period based on reports from the scientific staff. Feedback from laboratory scientists was that the process was not disruptive or overly burdensome.

In this study, the only change to the blood culture workflow was the use of pre-warmed chocolate agar plates, while the inoculation, incubation (at least 2 h in 5% CO_2_ at 37 °C) and preparation for MS identification remained the same. The same instrument for identification was used throughout the study period without database changes. As such, it appears that achieving plate temperature of ~36 °C for the duration of incubation is an important factor for short durations of culture.

It is interesting to note the slight increase in the rate of errors between the baseline and interventional periods. It is notable (Table 2) that most of the incorrect organisms identified with high confidence (>99%) were coagulase-negative staphylococci (except for a single instance of *Staphylococcus aureus*). There are many reasons for incorrect results from MALDI-TOF MS, including contamination of the MS plates with other specimens or the *Escherichia coli* control spot, incorrect specimen inoculation or the recording of a low discriminating identification, which should potentially be discounted as an inaccurate result.

This study has some important limitations. Firstly, the study was a comparison with a previous retrospective baseline, rather than a parallel sequence of investigation. This was a pragmatic choice as the requirement for parallel testing (one blood culture requiring two choc drops to be set up, incubated and identified) would be burdensome and more time/resource consuming. This may lead to other factors influencing the outcome, including organism load and time to positivity, which were not controlled for in this study. There may also be seasonal variation in rates of bacterial bloodstream infection, such as *Staphylococcus aureus*/*Streptococcus pneumoniae*, increasing during influenza seasons [14].

Secondly, this study was performed at a single centre in a medium-sized laboratory, which may limit its generalization to higher throughput settings. However, it must also be noted that the laboratory receives most of its blood cultures from tertiary centre referral hospitals, which includes medical, surgical, haematology/oncology and intensive care disciplines; therefore, a representative sample of a broad range of potential organisms should be expected and was demonstrated in this study.

Thirdly, there was limited ability to assess the potential for improvements in time to correct identification (which is an important factor when it comes to appropriate antibiotic selection) as well as the potential positive impact any change has on clinical outcomes. This was partly due to this study being focused on a laboratory change rather than clinical outcomes, and partly due to the limitations of the current workflow with the choc drop being performed twice a day rather than a set period post-inoculation for each individual positive blood culture. Additionally, due to workflow constraints, it was not possible to guarantee the exact time that the choc drop was performed. The responsible scientist would perform a choc drop on any plate that had at least 2 h of incubation, but this period could be significantly longer, up to a maximum of 6–8 h of incubation.

## Conclusion

This hypothesis-generating study demonstrates that a low-impact, low-cost intervention of pre-warmed chocolate agar significantly increased the proportion of correct, high-confidence identification with minimal change in laboratory workflow and a small and acceptable rate of errors. This is supported by a biologically plausible mechanism, as temperature is a known factor for better bacterial growth and variability in time for a plate to achieve target temperature has been previously demonstrated experimentally [9].

As blood cultures remain the gold standard investigation for sepsis, it would be important to confirm these findings with further studies, designed around observing choc drops set up in parallel on pre-warmed and non-pre-warmed agar. The aims would be to observe if there were any advantages to the modification of this method, including a more rapid, accurate preliminary identification, a significant difference in distinguishing Gram-positive and Gram-negative organisms and impact on laboratory workflows and clinical outcomes.

## Supplementary material

10.1099/jmm.0.002057 Uncited Table S1.

## Abbreviations

CI: confidence interval
MALDI-TOF MS: matrix-assisted laser desorption/ionization time-of-flight MS
