# Supplementary material for: Hot chocolate: pre-warming chocolate agar improves correct organism identification from choc drop by 16.1%
Source: J Med Microbiol. 2025 Sep 2;74(9):002057. doi: 10.1099/jmm.0.002057 (PMC12451755; doi:10.1099/jmm.0.002057)
Supplement: Uncited Table S1. [file jmm-74-02057-s001.pdf]

Supplementary Table 1

Table of organisms encountered in the baseline and interventional period

Organisms Isolated- No choc drop identification		
	**Baseline**	**Interventional**
**Gram positive:** **N (%)**	*Staphylococcus epidermidis* 50 (24.6%)	*Staphylococcus epidermidis* 15 (16.9%)
	Staphylococcus aureus 15 (7.4%)	*Staphylococcus aureus* 8 (9.0%
	Staphylococcus capitis 15 (7.4%)	*Enterococcus faecium* 6 (6.7%)
	Staphylococcus hominis 15 (7.4%)	*Staphylococcus capitis* 5 (5.60%)
	Paenibacillus sp. 7 (3.5%)	*Staphylococcus hominis* 4 (4.5%)
	Bacillus sp. 6 (3.5%)	*Micrococcus luteus* 3 (3.4%)
	Micrococcus luteus 5 (2.5%)	*Streptococcus anginosus* 3 (3.4%)
	Staphylococcus haemolyticus 5 (2.5%)	*Parvimonas micra* 2 (2.2%)
	Clostridium sp. 4 (2.0%)	*Propionibacterium acnes* 2 (2.2%)
	Staphylococcus warneri 4 (2.0%)	*Bacillus* sp. 1 (1.1%)
	Staphylococcus lugdunensis 3 (1.5%)	*Corynebacterium* sp. 1 (1.1%)
	Streptococcus intermedius 3 (1.5%)	*Leuconostoc* sp. 1 (1.1%)
	Streptococcus mitis/oralis 3 (1.5%)	*Paenibacillus* sp. 1 (1.1%)
	Actinomyces sp. 1 (0.5%)	*Staphylococcus auricularis* 1 (1.1%)

	Parvimonas micra 2 (1.0%)	*Staphylococcus pasteuri* 1 (1.1%)
	Staphylococcus cohnii 2 (1.0%)	*Streptococcus intermedius* 1 (1.1%)
	Streptococcus agalactiae 2 (1.0%)	*Streptococcus mitis/oralis* 1 (1.1%)
	Streptococcus constellatus 2 (1.0%)	*Streptococcus pneumoniae* 1 (1.1%)
	Streptococcus pyogenes 2 (1.0%)	*Streptococcus pyogenes* 1 (1.1%)
	Corynebacterium sp. 1 (0.5%)	*Streptococcus salivarius* 1 (1.1%)
	Dermacoccus sp 1 (0.5%)	*Streptococcus sanguinis* 1 (1.1%)
	Peptoniphilus asaccharolyticus 1 (0.5%)	
	Prevotella bivia 1 (0.5%)	
	Streptococcus cristatus 1 (0.5%)	
	Streptococcus dysgalactiae subsp equisimilis 1 (0.5%)	
	Streptococcus viridans group 1 (0.5%)	
**Gram negative: N (%)**	*Escherichia coli* 12 (5.9%)	*Escherichia coli* 9 (10.1%)
	Enterobacter cloacae complex 4 (2%)	*Bacteroides* sp. 5 (5.6%)
	Bacteroides fragilis 3 (1.5%)	*Bacteroides fragilis* 4 (4.5%)
	Serratia marcescens 3 (1.5%)	*Enterobacter cloacae* complex 2 (2.2%)

	Campylobacter jejuni 2 (1.0%)	*Pseudomonas aeruginosa* 2 (2.2%)
	Klebsiella pneumoniae 2 (1.0%)	*Campylobacter jejuni* 1 (1.1%)
	Burkholderia cepacia complex 1 (0.5%)	*Desulfovibrio* sp. 1 (1.1%)
	Fusobacterium necrophorum 1 (0.5%)	*Klebsiella pneumoniae* 1 (1.1%)
	Haemophilus parainfluenzae 1 (0.5%)	*Weeksella virosa* 1 (1.1%)
	Providentia stuartii 1 (0.5%)	
	Pseudomonas aeruginosa 1 (0.5%)	
	Pseudomonas stutzerii 1 (0.5%)	
	Stenotrophomonas maltophilia 1 (0.5%)	
**Yeast:** **N (%)**	*Candida tropicalis 10* (4.9%)	*Candida glabrata* 2 (2.2%)
	Candida albicans 2 (1.0%)	*Candida parapsilosis* 2 (2.2%)
	Candida glabrata 1 (0.5%)	
	Other *Candida* sp. 1 (0.5%)	
	Pichia kudriavzevii 1 (0.5%)	
Organisms Isolated- High Confidence Identification	**Baseline**	**Interventional**
**Gram positive:** **N (%)**	*Staphylococcus aureus* 68 (24.8%)	*Staphylococcus aureus* 62 (21.8%)
	Staphylococcus epidermidis 16 (5.8%)	*Staphylococcus epidermidis* 28 (9.9%)

	Staphylococcus hominis 14 (5.1%)	*Staphylococcus hominis* 15 (5.3%)
	Enterococcus faecalis 8 (2.9%)	*Enterococcus faecium* 10 (3.5%)
	Paenibacillus sp. 6 (2.2%)	*Staphylococcus capitis* 8 (2.8%)
	Streptococcus agalactiae 6 (2.2%)	*Enterococcus faecalis* 5 (1.8%)
	Micrococcus luteus 4 (1.5%)	*Staphylococcus haemolyticus* 5 (1.8%)
	Staphylococcus capitis 4 (1.5%)	*Streptococcus mitis/oralis* 5 (1.8%)
	Staphylococcus haemolyticus 4 (1.5%)	*Streptococcus pneumoniae* 3 (1.1%)
	Enterococcus faecium 3 (1.1%)	*Bacillus* sp. 2 (0.7%)
	Streptococcus pneumoniae 3 (1.1%)	*Staphylococcus warneri* 2 (0.7%)
	Bacillus sp. 2 (0.7%)	*Streptococcus agalactiae* 2 (0.7%)
	Staphylococcus lugdenensis 2 (0.7%)	*Streptococcus gordonii* 2 (0.7%)
	Streptococcus anginosus 1 (0.4%)	*Streptococcus pyogenes* 2 (0.7%)
	Streptococcus dysgalactiae subsp equisimilis 1 (0.4%)	*Clostridium tertium* 1 (0.4%)
	Streptococcus parasanguinis 1 (0.4%)	*Propionibacterium acnes* 1 (0.4%)
	Streptococcus pyogenes 1 (0.4%)	*Paenibacillus* sp. 1 (0.4%)
	Streptococcus sanguinis 1 (0.4%)	*Peribacillus simplex* 1 (0.4%)

		Staphylococcus kloosii 1 (0.4%)
		Streptococcus dysgalactiae 1 (0.4%)
**Gram negative: N (%)**	*Escherichia coli* 70 (25.6%)	*Escherichia coli* 77 (27.1%)
	Klebsiella pneumoniae 26 (9.5%)	*Enterobacter cloacae* complex 15 (5.3%)
	Pseudomonas aeruginosa 5 (1.8%)	*Klebsiella pneumoniae* 10 (3.5%)
	Serratia marcescens 4 (1.5%)	*Pseudomonas aeruginosa* 10 (3.5%)
	Salmonella typhi 3 (1.1%)	*Serratia marcescens* 3 (1.0%)
	Stenotrophomonas maltophilia 3 (1.1%)	*Klebsiella oxytoca* 2 (0.7%)
	Klebsiella oxytoca 2 (0.7%)	*Acinetobacter lwoffii* 1 (0.4%)
	Proteus mirabilis 2 (0.7%)	*Citrobacter koseri* 1 (0.4%)
	Campylobacter jejuni 1 (0.4%)	*Haemophilus influenzae* 1 (0.4%)
	Citrobacter koseri 1 (0.4%)	*Morganella morganii* 1 (0.4%)
	Enterobacter cloacae complex 1 (0.4%)	*Stenotrophomonas maltophilia* 1 (0.4%)
	Morganella morganii 1 (0.4%)	
	Salmonella species (non-typhi) 1 (0.4%)	
**Yeast: N (%)**	*Pichia kudriavezevii* 5 (1.82%)	
